# Facilitating HIV status disclosure for pregnant women and partners in rural Kenya: a qualitative study

**DOI:** 10.1186/1471-2458-13-1115

**Published:** 2013-12-02

**Authors:** Melonie M Walcott, Abigail M Hatcher, Zachary Kwena, Janet M Turan

**Affiliations:** 1 University of Alabama at Birmingham, 1530 3rd Ave S, Birmingham, AL 35294, USA; 2 University of California, San Francisco, 50 Beale Street, San Francisco, CA 94105, USA; 3 Wits Reproductive Health & HIV Institute, University of the Witwatersrand, 22 Esselen Street, Johannesburg 2001, South Africa; 4 Research Care and Treatment Program, Kenya Medical Research Institute, Kisumu, Kenya; 5 Department of Health Care Organization and Policy, University of Alabama at Birmingham, RPHB 330, 1530 3rd Ave S, Birmingham, AL 35294, USA

**Keywords:** Facilitated HIV disclosure, PMTCT, Home-visits, Pregnant women

## Abstract

**Background:**

Women’s ability to safely disclose their HIV-positive status to male partners is essential for uptake and continued use of prevention of mother-to-child transmission (PMTCT) services. However, little is known about the acceptability of potential approaches for facilitating partner disclosure. To lay the groundwork for developing an intervention, we conducted formative qualitative research to elicit feedback on three approaches for safe HIV disclosure for pregnant women and male partners in rural Kenya.

**Methods:**

This qualitative acceptability research included in-depth interviews with HIV-infected pregnant women (n = 20) and male partners of HIV-infected women (n = 20) as well as two focus groups with service providers (n = 16). The participants were recruited at health care facilities in two communities in rural Nyanza Province, Kenya, during the period June to November 2011. Data were managed in NVivo 9 and analyzed using a framework approach, drawing on grounded theory.

**Results:**

We found that facilitating HIV disclosure is acceptable in this context, but that individual participants have varying expectations depending on their personal situation. Many participants displayed a strong preference for couples HIV counseling and testing (CHCT) with mutual disclosure facilitated by a trained health worker. Home-based approaches and programs in which pregnant women are asked to bring their partners to the healthcare facility were equally favored. Participants felt that home-based CHCT would be acceptable for this rural setting, but special attention must be paid to how this service is introduced in the community, training of the health workers who will conduct the home visits, and confidentiality.

**Conclusion:**

Pregnant couples should be given different options for assistance with HIV disclosure. Home-based CHCT could serve as an acceptable method to assist women and men with safe disclosure of HIV status. These findings can inform the design and implementation of programs geared at promoting HIV disclosure among pregnant women and partners, especially in the home-setting.

## Background

HIV status disclosure is essential to reduce transmission of HIV to sexual partners [1], particularly among populations with high rates of sero-status discordancy [2]. The prevalence of couple HIV sero-discordance in sub-Saharan Africa is very high [3], with married and cohabitating couples being disproportionately affected [4].

Disclosure may be particularly crucial for pregnant women. During pregnancy, women are at considerably increased risk for HIV acquisition (male-to-female) and transmission (female-to-male) [5,6]. Without partner support, it is often difficult for women to adhere to recommended HIV treatment and breastfeeding regimens, behaviors that are necessary to reduce transmission of HIV to their infants, protect their own health, and ensure the health of their partner [7-11].

Studies have shown that when male partners are involved in HIV testing and antenatal care (ANC), women are more likely to accept antiretroviral (ARV) prophylaxis [12-15], give birth in a health facility [16], and adhere to recommended HIV-related care [17]. When women have disclosed their HIV status and male partners are involved in antenatal care, HIV-free infant survival improves [18,19]. Thus, testing women and their partners during pregnancy, along with support for mutual disclosure, could prevent HIV transmission both vertically and horizontally [14,20,21]. Despite this, most PMTCT programs test pregnant women alone at the antenatal clinic and offer little support for facilitating safe disclosure of an HIV-positive diagnosis to male partners [22,23].

After testing for HIV, many women do not feel safe and comfortable disclosing their HIV status to partners due to fears of partner reactions to an HIV-positive test result [24,25]. These anticipated consequences, which are well documented in the literature, include fears of accusations of infidelity [1,8], abandonment [8,26], violence [8,27,28], stigma and discrimination [26,29-31], separation [32], and fear of loss of economic support [7,8,10]. It is therefore not surprising that although rates of HIV disclosure in sub-Saharan Africa range from 17%-86%, lower rates are observed among women who are tested for HIV in ANC settings [8].

In facilitated disclosure (also called assisted disclosure, dual disclosure, or counselor-supported disclosure), a counselor is present during the disclosure process and provides information and support [33]. Facilitated disclosure has the potential to increase support and engagement in HIV care, without increasing risk of adverse social events. Home-based counseling and HIV testing (HBCT) is another strategy used to expand access to couple HIV testing services in sub-Saharan Africa, and it includes support for mutual HIV status disclosure. Studies in Kenya and elsewhere in Africa have found that HBCT is feasible, acceptable, and cost-effective [30,34,35], and it has been suggested that HBCT could increase couples’ involvement in HIV testing and reduce HIV transmission among discordant couples [34]. However, little is known about HBCT specifically for pregnant couples, or how facilitated disclosure may enhance service utilization and health outcomes for pregnant women and their families. Similarly, little is known about the disclosure process, making it challenging to develop targeted programs for this purpose.

In order to address this gap we conducted formative qualitative research to understand the disclosure process and assess the acceptability of a home-based approach for supporting HIV counseling and testing and mutual status disclosure among HIV-infected pregnant women, their male partners, and service providers in rural Kenya.

## Methods

### Setting and context

The prevalence of HIV among adults in Kenya is approximately 7.4% [1,36,37], with women being disproportionately affected [38]. Nyanza Province has the highest HIV prevalence and infant mortality rate in Kenya [39,40]. Among pregnant women in Nyanza, HIV prevalence as high as 20.7% in ANC settings has been documented [40]. This study was conducted in two communities in rural Nyanza Province, Kenya, during the period June to November 2011, as part of the intervention development phase of the Maternity in Migori and AIDS Stigma (MAMAS) Study (NIMH # K01MH081777). The MAMAS Study was a prospective mixed-methods study designed to assess the effects of HIV-related stigma on pregnant women’s use of health services. Study sites were government health facilities that are supported by the President’s Emergency Plan for AIDS Relief (PEPFAR)-funded Family AIDS Care and Education Services (FACES) program [31].

### Data collection

This formative qualitative research included in-depth interviews with HIV-infected pregnant women (n = 20) and male partners of HIV-infected women (n = 20) as well as two focus groups with service providers (n = 16). HIV-positive pregnant women were purposively recruited from four antenatal clinics at local government primary healthcare facilities to capture variation in socio-demographics and partner disclosure status, based on information in clinic records. Male partners of HIV-positive pregnant women were recruited via HIV-positive women who attended the ANC clinics. To avoid unwanted disclosure and potential negative consequences, men had to already be aware of their female partner’s HIV-positive status in order to be recruited for the study and permission was obtained from the women before their partners were invited to participate in the study. Service providers recruited for the study included health professionals, community health workers, and other community service providers in the two selected study communities. Providers were purposively selected to obtain a range of those who have experience working with HIV-positive clients in the clinic and the community, and included clinic-based lay health workers, community health workers, clinicians, social workers/counselors, religious leaders, and local government representatives. Participants in this study were 18 years of age and older and were given a reimbursement of 400 Kenyan Shillings (an equivalent of around US$5.00) for any travel expenses incurred related to their participation in this study.

In-depth interview and focus group discussion guides were developed by the research team. The topics that were identified for exploration included: 1) barriers and facilitators to HIV disclosure, 2) male partner involvement in antenatal care and HIV testing, 3) disclosure of HIV status within a couple, 4) perceived advantages and disadvantages of facilitated disclosure for couples, and 5) different approaches for assisting pregnant women and their partners with disclosure as described below:

1. The health worker gives the client who tested HIV-positive advice on how to share his/her HIV status with the partner. Afterwards the client shares his/her HIV status with the partner on his/her own.

2. The health worker asks the client to bring the partner to the health facility for assisted sharing of the HIV test results and couple counseling.

3. The health worker comes to the house for a home visit with the client and the partner for sharing HIV status and couple counseling.

Participants were also asked about their views on three methods that could be used to assist a person who has tested HIV-positive with disclosure to his/her partner, namely: 1) disclosure by the health worker to the partner, 2) disclosure to the partner by the person who tested HIV-positive in the presence of the health worker, and 3) the health worker encourages the couple to get tested together.

A brief form was used to collect socio-demographic information about the participants. The interviews and focus group discussions were conducted by trained interviewers/focus group moderators in the local language (Dholuo) in the communities and generally lasted 1–2 hours. All the interviews and discussions were audio-recorded after obtaining permission from the participants. The audio-recordings were transcribed verbatim and translated into English by experienced translators based in Kenya and names or other identifying information were not included in the transcripts. A summary report was prepared after conducting each interview to document emerging themes and clarify uncertainties. Ethical approval was granted by University of California San Francisco, the Kenya Medical Research Institute Ethical Review Committee, and the University of Alabama at Birmingham. All participants provided written (in-depth interview participants) or verbal (focus group participants) informed consent before they were enrolled in the study.

### Analytic methods

Qualitative data were coded and analyzed using a framework approach [41], also drawing on grounded theory methods. Data were managed in the NVivo 9 software (QSR International Pty Ltd, Doncaster, Victoria, Australia). Two members of the research team read the transcripts to become familiar with the data and developed the codebook to facilitate analysis. Initially broad pre-defined codes were developed based on the topics covered in the interview and focus group discussion guides, and emerging themes in the transcripts. The codes were specifically defined and the coding framework was agreed on by the research team members before the transcripts were coded. The coded data were then reviewed by the research team, and fine codes were developed based on the initial reports generated from the broad coding. The broad themes were then recoded using the fine codes to further categorize the data. These were then used to produce a detailed analytical report, which examined key themes and findings and included illustrative quotations from the database. Query tools in NVivo were used to further explore the data, assist in identifying emerging patterns and themes, and make comparisons between categories and sub-groups.

## Results

### Socio-demographic and HIV-related characteristics

Fifty-six participants (20 HIV-positive pregnant women, 20 male partners of HIV-positive pregnant women, and 16 service providers) aged 19–69 years (33.4 ± 10.8) participated in an interview or a focus group discussion. The participants’ spouses also had a similar age range (19–70; 33.4 ± 10.8). Most of the participants had at least primary level education, and many service providers had some form of college-level training. Apart from the service providers the participants primarily worked in agriculture, business (include selling food items), or were semi-skilled workers (driver, tailor, painter, welder). Most of the participants were married (66% monogamous and 23% polygamous). However, only 70% of the participants were currently living with their spouse. Most of the participants (53%) had 3 or more living children. Of the participants who chose to share information about their HIV-positive status (pregnant women and male partners), most (60%) had been diagnosed within the last 4 years and 75% had disclosed their status to their sexual partners (Table 1).

**Table 1:** Socio-demographic and HIV-related characteristics of participants

**Characteristics**	**Total N (%)**	**Pregnant women N = 20**	**Male partners N = 20**	**Health workers N = 16**
**Participant age**				
18-24	13 (23.2)	8 (40.0)	3 (15.0)	2 (12.5)
25-34	23 (41.1)	10 (50.0)	7 (35.0)	6 (37.5)
35-44	12 (21.4)	2 (10.0)	7 (35.0)	3 (18.8)
≥45	8 (14.2)	0 (0.0)	3 (15.0)	5 (31.3)
**Spouse age**				
18-24	7 (12.5)	3 (15.0)	4 (20.0)	0 (0.0)
25-34	20 (35.7)	6 (30.0)	4 (25.0)	4 (25.0)
35-44	13 (23.2)	7 (35.0)	2 (10.0)	4 (25.0)
≥45	10 (17.9)	6 (37.5)	0 (0.0)	6 (37.5)
**Participant education**				
Did not complete primary	15 (26.8)	5 (25.0)	9 (45.0)	1 (6.3)
Completed primary	17 (30.4)	11 (55.0)	4 (20.0)	2 (12.5)
Did not complete secondary	5 (8.9)	1 (5.0)	3 (15.0)	1 (6.3)
Complete secondary	10 (17.9)	2 (10.0)	4 (20.0)	4 (25.0)
Any college	9 (16.1)	1 (5.0)	0 (0.0)	8 (50.0)
**Spouse education**				
Did not complete primary	20 (35.7)	7 (35.0)	10 (50.0)	5 (31.3)
Completed primary	19 (33.9)	8 (40.0)	8 (40.0)	1 (6.3)
Did not complete secondary	1 (1.8)	0 (0.0)	0 (0.0)	0 (0.0)
Complete secondary	8 (14.3)	3 (15.0)	2 (10.0)	3 (18.8)
Any college	6 (10.7)	1 (5.0)	0 (0.0)	5 (31.3)
**Marital status**				
Monogamous marriage	37 (66.1)	11 (55.0)	13 (65)	13 (81.3)
Polygamous marriage	13 (23.2)	5 (25.0)	7 (35.0)	1 (6.3)
Single	3 (5.4)	1 (5.0)	0 (0)	2 (12.5)
Widow	3 (5.4)	3 (15.0)	0 (0)	0 (0.0)
**Currently living with spouse**	39 (69.6)	10 (50.0)	18 (90.0)	11 (68.8)
**Occupation**				
Agriculture	11 (19.6)	1 (5.0)	10 (50.)	0 (0.0)
Business/sales	7 (12.5)	5 (25.0)	2 (10.0)	0 (0.0)
Health/community services	15 (26.8)	0 (0.0)	0 (0.0)	14 (87.5)
Skilled worker	11 (19.6)	3 (15.0)	8 (40.0)	0 (0.0)
Housewife	9 (16.1)	9 (45.0)	0 (0.0)	0 (0.0)
Retired	1 (1.8)	0 (0.0)	0 (0.0)	1 (6.3)
**Length of time in current job (years)**				
< 1	2 (3.6)	1 (5.0)	1 (5.0)	0 (0.0)
1-3	13 (23.2)	3 (15.0)	4 (20.0)	6 (37.5)
4-6	11 (19.6)	4 (20.0)	4 (20.0)	3 (18.8)
≥7	18 (32.1)	2 (10.0)	11 (55.0)	5 (31.3)
**Number of living children**				
0	8 (14.3)	4 (20.0)	2 (10.0)	2 (12.5)
1	5 (8.9)	2 (10.0)	1 (5.0)	2 (12.5)
2	13 (23.2)	5 (25.0)	4 (20.0)	4 (25.0)
3 or more	30 (53.6)	9 (45.0)	13 (65.0)	8 (50.0)
**HIV status of participants**				
Positive	36 (90.0)	20 (100.0)	16 (80.0)	Not asked
**Year of HIV diagnosis**				
More than 4 years ago	6 (15)	1 (5.0)	5 (25.0)	
Within the past 4 years	24 (60)	16 (80.0)	8 (40.0)	
**Disclosed HIV status to partner**	30 (75)	14 (70)	16 (80.0)	Not asked
**Partner tested for HIV**				Not asked
Yes	31 (77.5)	11 (55.0)	20 (100.0)	
No/Do not know	9 (22.5)	9 (45.0)	0 (0.0)	
**Knew HV status before getting pregnant**	20 (50.0)	9 (45.0)	11 (55.0)	Not asked
**Pregnancy duration (weeks)**			Not asked	Not asked
3– 28	12 (60.0)	12 (60.0)		
29 – 40	7 (35.0)	7 (35.0)		

### Barriers and facilitators to HIV disclosure among couples

Barriers to HIV status disclosure among couples included fears of abuse, disharmony in the relationship, and stigma. The participants explained that fears of abuse—including being blamed for the infection, break-up of the relationship, and bodily harm—served as contributing factors to non-disclosure of one’s HIV status. While men feared being accused of infidelity or being promiscuous, women tended to fear being blamed for bringing the infection into the home:

“*The person who gets tested first and takes this thing to the home is viewed as the person who brought this disease and this brings the quarrel*”. (39-year-old male partner)

As described by one pregnant woman, disclosure sometimes leads to violence from a male partner, particularly in the case of a pregnant woman requesting her male partner to get tested for HIV following her antenatal visit:

“*The problem is when the husband is too cruel or high tempered. The moment you start sharing with him, he tells you to shut up and gives you threats like he will kill you, or accuses you of infecting him. This is after knowing your status, when you are telling him to go for HIV test*”. (39-year-old pregnant woman)

Participants described disharmony between partners—including lack of love, trust, and understanding—as a significant barrier to HIV disclosure. Participants reported that these factors promote secrecy, create tension and distancing in the relationship, thus making it difficult to initiate a conversation about HIV disclosure:

“*If there is mistrust in the house, there will be no communication in that house because you cannot share with me something and you do not trust me and myself I will do the same to you if you do not trust me*”. (31-year-old male partner)

Participants also struggled with thoughts of how they will be viewed by the public after disclosing their HIV status. Being infected with HIV was perceived as an embarrassing event, distinct from other illnesses like malaria, and associated with a great deal of shame:

*“People don’t want to be looked down upon. In case it was malaria, they could gladly accept to tell others. In my village, people think that that being [HIV-] infected is shameful, hence they will not discuss it easily as expected. Some people will therefore avoid anyone meddling into their HIV status since they believe that it is very shameful”.* (52-year-old male partner)

Thus, some individuals described non-disclosure as a strategy to avoid community stigma:

*“I think there is somewhere that the fears to disclose to the wife because the wife will tell other people. I think what’s happening with the man is just stigma”.* (24- year-old service provider)

Other barriers that were identified include the need to protect the partner, denial and poverty. The need to protect one’s partner was expressed by a few male participants who stated that they did not disclose because they believe their female partner could not handle the information. One man expressed that he chose not to disclose, due to concerns that the information would be too overwhelming for his partner:

“*I didn’t tell her, because being a woman she is comparatively weaker than a man and this disclosure was likely to give her considerable stress which was obviously seen when her child died and at the same time I was bedridden. I knew she could not handle it*”. (40-year-old male partner)

Poverty, including insufficient food and other urgent material needs, was also mentioned as a reason for avoiding HIV status disclosure. Participants described the importance of meeting basic subsistence needs prior to disclosing their status to partners:

“*You only disclose after knowing how you will survive*”. (20-year-old pregnant woman)

Poverty was a particularly important consideration for pregnant women since male partners might withdraw their financial support:

*“Another thing is economical issue, you realize that in the rural set up most people are the sole bread winners and mostly it’s the male and the spouse has been tested and realizes that she is HIV-positive she fears disclosing because she might not get the support from the man”.* (34-year-old male health worker)

When participants were asked what things make it easier for individuals to share their HIV status, most expressed the need for a candid open relationship with one’s partner. Being open about the infection with one’s partner was also seen as a first step to develop the courage to access HIV care. The need to be “free” with one’s partner and share the HIV diagnosis was consistently mentioned especially among male partners. Additionally, HIV disclosure was seen by some as a unifying force in the household, allowing for mutual support around medical decisions like clinic attendance and adherence:

“*What can help men to talk to their women about their HIV status is for men to look at it as something that is going to unite them in their house. If they talk about it in the right and peaceful manner in their house, that is one thing that can unite them in their house. One of the good things about talking to your wife about your HIV status is that these women can remind them to swallow their medicine and also the date of attending, adherence, it becomes their family responsibility*”. (39- year-old male partner)

However, participants (especially females) also cautioned that before disclosing, steps should be taken to create an environment that is likely to foster a favorable reaction. This includes preparing a nice meal, pampering one’s partner, and being polite. The participants also explained that the psychological well-being of the discloser and the partner must be taken into consideration before disclosing:

“*You must think before disclosing. You must check his mood and in case he was annoyed sometimes back, do not disclose. In case he is happy, look for a time and talk to him about it*” (22-year-old pregnant women)

### Perceptions of “facilitated disclosure”

Overall, the idea of facilitated disclosure was highly favored among participants who saw this as a beneficial service. Some of the perceived benefits of facilitated disclosure expressed by the participants were: improved ability to accept HIV positive results, increased knowledge about the illness and HIV medication, contributions to adherence, and reduced fears related to HIV infection due to information and counseling given by the health worker.

Participants highlighted that facilitated disclosure could foster understanding between partners, which they felt was essential to build trust and effectively communicate about one’s HIV test result:

“*It is good because the doctor counsels both of you so that you can understand him better so that you don’t harbor hatred in your hearts but feel happy. You say that ‘even though this is our status as per now, we are going to live according to the doctor’s advice.’ It brings a good message that you should not quarrel and that you should live at peace. My opinion is that the health provider will make the couples accept their status*”. (32-year-old pregnant woman)

In this quote, the pregnant woman describes a trust in health care providers’ ability to promote acceptance of status and a conflict-free response by partners. Service providers also highlighted that facilitated disclosure could speed the process of linkage to HIV care:

“*It’s also a benefit because it leads to early adherence as compared to self-disclosure which takes time, because they’ll know what to do after the test*”. (30- year-old service provider)

Although a number of participants reported that they did not foresee any disadvantages to facilitated disclosure, some participants expressed that even facilitated HIV status disclosure could still result in experiences of shock, separation, violence, and conflict in the home, so it needs to be handled with caution.

### Approaches for facilitated HIV disclosure services

The participants’ views on the advantages and disadvantages of the three approaches for facilitated disclosure services are summarized in Table 2. The approaches: “Doctor tells you to go and bring your partner to the health care facility” and “Doctor decides to visit the home”, were almost equally favored by the participants.

**Table 2:** Participants views on the advantages and disadvantages of 3 methods for delivery of facilitated disclosure

**Facilitated disclosure method**	**Advantages**	**Disadvantages**
Health worker teaches you how to go and talk to your spouse	1.Given opportunity to personally disclose HIV to partner (open up to partner)	1. Difficult to discuss HIV status with partner alone
	2. Added knowledge which can initiate HIV disclosure	2. May result in disagreement and quarrels between couples
	3. Increase understanding between couples	3. May result in abuse
	4. Learn strategies for HIV disclosure	4. May result in separation
Health worker tells you to go and bring your partner to health care facility	1. Opportunity to be counseled together	1. Partner may refuse to go to clinic
	2. Shows doctors care about couples’ lives	2. Inviting partner to go to health care facility for HIV testing could evoke suspicion of positive HIV status which could result in abuse/violence
	3. Learn how to disclose to partner	3. May be blamed for the HIV infection
	4. Support is provided to assist with disclosure	
	5. Increase understanding between couples	
	6. Increase communication between couples	
	7. Could contribute to PMTCT	
	8. Ability to access treatment	
	9. Privacy	
Health worker decides to visit the home	1. Contribute to acceptance of HIV infection	1. May spark questions/concerns or gossip in the community
	2. Contribute to adherence to medication	2. Community members may suspect HIV-infection
	3. Increase knowledge	3. May result in stigma
	4. Reduce cost to go the health care facility cost	4. May experience embarrassment for self or family
	5. Reduce hospital fears	5. Potential breach of confidentiality by health workers
	6. Potential for individuals in the community to benefit	6. Neighbors may become knowledgeable of one's HIV status
	7. Potential to have health checks for self and family at home	7. May result in separation
	8. Shows doctors care about couples’ lives	8. May experience abuse/violence from partner
	9. Promotes HIV testing	9. Some community members may avoid the home visit

Participants expressed that disclosure at health facilities provided an opportunity to be educated, counseled, and tested for HIV with their partners, and provided direct access to treatment and care. This they believed could contribute to improved understanding and communication between couples. Despite the benefits of this option, some female participants were mindful that their partners may not respond positively to the invitation to come to the health facility. Thus, telling one’s partner to go the clinic does not guarantee their involvement:

“*If I am educated about it, I will talk to him. But, majority of them don’t listen. Let me take myself as an example, I was taught how to talk to him and counsel him but it never worked out*”. (30-year-old pregnant woman)

Disclosure via home visit was considered beneficial as it provided an opportunity for couples to be tested together in their homes. Additionally, service providers noted that a home visit reduced cost for clients and would circumvent fears associated with going to the hospital:

“*I think this kind of method is cheaper, because as I rest in my home, I can be surprised by visitors visiting my home at the gate to do for me counseling. It is good because it will reduce for me the cost of transportation, when bringing women to the health care facility, if sometimes you are a polygamous man with four wives*”. (35-year-old service provider)

Participants noted that this method appears to be particularly helpful for individuals who are sick, as these individuals may not have the strength or resources to visit health care facilities.

Pregnant women suggested that couples should receive prior notice of the home visit, especially the male partner. In general, participants tended to believe their partners were likely to prefer the same method that they themselves preferred.

Although a home visit was primarily seen as being beneficial, a number of potential consequences and disadvantages were highlighted (Table 2). These consequences seem to stem from the heightened curiosity created in the community when a healthcare worker visits an individual’s home. Participants noted that neighbors may assume that those persons receiving home visits are those taking HIV medication or affected by HIV. Participants reported that neighbors may invite themselves over if a health care worker were to visit the home, in an effort to ascertain the reason for the visit:

“*The greatest thing is embarrassment because it will look like you are the one who has AIDS or your entire home has it. Your children will also know that their parents are in such a state and that is why this people have come. This can create disturbance*”. (48-year-old male partner)

Participants also expressed concern about the potential impact of obtaining discordant results during a home visit, especially if the woman’s result is HIV-positive and the man’s is HIV-negative. Women in this setting were seen as especially vulnerable to forms of intimate partner violence, such as forced exile from the home:

“*In case the results come out different, you will fall in trouble. However, if the results come out the same, things might cool down. It will be worse if the man turns negative and the woman positive. He may decide to chase you away*”. (30-year-old pregnant women)

It was believed that women who lived within an extended family setting would be likely to be chased away due to pressure from in-laws. Because of these gender dynamics, men in our sample seemed more comfortable with home visits than women.

Most participants did not support the disclosure option in which the “the doctor tells the client how to disclose to their partner”. The participants shared that personal disclosure to their partners requires openness in the relationship, or “being free”. The main limitations of this method are fears of being blamed for the infection and inherent difficulties in finding the courage to disclose one’s HIV status. This fear of disclosure was particularly pronounced among women in polygamous relationships:

“*This approach is so difficult and it is disturbing many people, both men and women. For us we are three women and if I am the one who went for HIV testing, it’s difficult to gather people together, and share that information. It is the reason for you to be beaten up, because you do not know the tension of the other person*”. (30-year-old pregnant women)

In this quote, the pregnant woman articulates the need to protect oneself (by avoiding disclosure) in order to avoid physical violence. This fear may be particularly salient for pregnant women, who perhaps feel that they are providing safety for themselves as well as for their unborn child.

### Methods that can be used during facilitated disclosure

Table 3 shows the advantages and disadvantages of the three methods that could be used in a facilitated disclosure session with a health worker: 1) health worker discloses the HIV-infected individual’s status, 2) HIV-infected individual discloses to his/her partner in the presence of the health worker, and 3) the couple is tested for HIV together (with the person who has already tested HIV-positive being retested as if he/she does not know the test result) followed by facilitated disclosure.

**Table 3:** Advantages and disadvantages of approaches that could be used during facilitated disclosure

**Option**	**Advantages**	**Disadvantages**
Health worker discloses HIV-infected status to partner		1. May result in blame for hiding infection
		2. Partner may become abusive
		3. Shows lack of communication in relationship
		4. Denies participants of their rights to disclose their HV status
		5. Partner will be shocked
		6. May cause disagreement
HIV-infected individual discloses to partner in Health worker presence	1. Provides protection (partner may not act violently in the presence of health worker)	1. May result in blame for hiding infection
	2. Partner can be encouraged to be tested for HIV	2. Partner may respond in shock
	3. Helps with the acceptance of the HIV diagnosis	3. May result in disagreement
	4. Can help to reduce transmission (partner will know to use condoms if having extramarital affair)	4. Fear others will hear about his/her HIV status
	5. Provides an opportunity to have questions answered	5. Could lead to separation
	6. Will be educated together	
	7. Promote understanding	
	8. Promote trust	
Partners are tested together	1. Learn about HIV together	1. May result in separation if discordant result
	2. Reduce blame	
	3. Improve support	
	4. Improve PMTCT	
	5. Reduce deceit	
	6. Improve understanding	
	7. Get to know HIV status together	
	8. Contribute to peace in the family	

Of the three methods, couple counseling and testing (Option 3) was preferred by most participants, primarily because even if an individual had prior knowledge of his/her HIV-positive diagnosis, they could opt for retesting with their partners without disclosing that they already knew they were HIV-positive. Participants expressed a strong desire for couple testing, be it at home or at a health facility, as this approach would significantly reduce the potential of being blamed for bringing the infection into the relationship:

“*If he [health worker] doesn’t disclose the result, no one will blame the other. You will understand if you are both tested and the results announced at the same time. However, you will be blamed to have brought it if you decide to go for the test alone. This will prevent quarrels*”. (38-year-old male partner)

The method in which the health worker discloses the HIV-infected individual status was least preferred among participants. For the most part the participants expressed that they did not like this approach and appeared to be uncomfortable that someone else was disclosing their HIV status for them. Expressions such as: “That is not a good thing”; “It isn’t a good move”; “It is just hard”, were used in reflecting their views of this option to facilitated HIV disclosure. The participants further explained that:

*“It is not good because he is going to be caught off guard with something that he did not know, yet you live together in the same house and he hears it from someone else”.* (22-year-old pregnant women)

### Home-based facilitated disclosure for pregnant women and male partners

**Figure 1:**
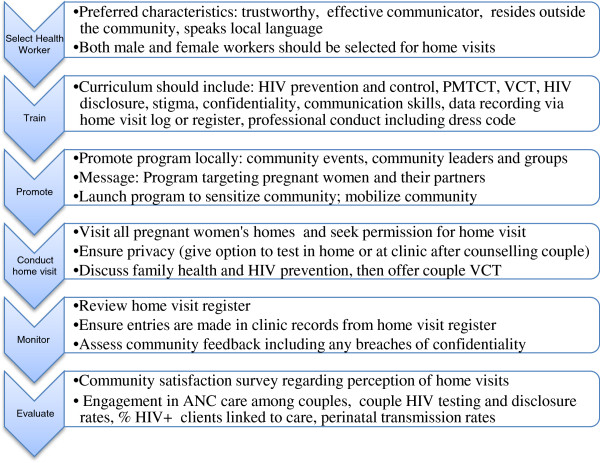
Home-based couple HIV counseling and testing with mutual disclosure - a research-informed approach.

Figure 1 summarizes recommendations from the study participants for implementing home-based HIV counseling and testing with mutual disclosure for pregnant women and male partners. Several themes emerged as important to ensure client satisfaction and safety in home visits (see Figure 1).

### Selection and training of health workers to conduct home visits

Participants explained that issues pertaining to trust and confidentiality are crucial to a home visit program. Desired characteristics for the health worker who will conduct the home visits included trustworthiness, effective communication skills, knowledge of local language, and prior training in HIV. Additionally, many participants recommended that the home visits should be conducted by middle-aged male and female health workers from outside the community. Participants expressed that such persons were more likely to be respected and trusted to keep health information confidential:

“*It’s easy if they come from another community than in your community, because people from the same place do not respect one another: ‘That so and so is the one doing such a thing, I will not visit that place - that has been disturbing many people and make them not go for HIV test. The health worker will gossip about them, and other will take the option of just not looking for that support but decide to die*”. (39-year-old pregnant woman)

Most of the participants preferred that a male and a female health worker conduct the visit together, as they believed HIV affects both sexes and this would increase the comfort level of both partners of the couple.

Participants emphasized the need for training of the healthcare workers to prepare them to effectively carry out their job in the community. Needs for training in HIV prevention and control, PMTCT, voluntary counseling and testing (VCT), HIV disclosure, stigma, confidentiality, communication skills, culture norms, and professional conduct (including dress code) were specified.

### Promotion of the program in the community

Participants explained the need for the program to be promoted in the community to sensitize individuals, reduce the risk of misunderstandings, and secure community support. When participants were asked what should be included in the community announcement promoting home visits, most reported that the content of the message should target pregnant women in general, not just those who are HIV-infected. This, they reported would reduce “branding” of the service as an “HIV service” and improve uptake of the service.

“*It is good to announce that the health worker is visiting the community to check on pregnant women without stating the main target group whether it’s for HIV pregnant women or HIV negative women*”. (28-year-old male partner)

“*I think that if they want to visit pregnant women living with HIV virus then this is what they should say while passing this information; that they are a health based organization and they have been sent to pay a visit to pregnant women to educate them on how they should live while pregnant and how to prevent diseases or complications that can arise during child birth*”. (39-year-old male partner)

Most of the participants reported that the best way to announce the program was via community events such as *barazas* (community meetings), churches, and schools. Other methods of promoting the program could include: worksite announcements, health care workers, social groups, media, posters and telephone. Participants expressed that the support from community leaders was crucial to the success of the program and that initial “buy-in” must be obtained from them.

### Conducting the home visit

Participants again stressed the importance of visiting all the homes in the community to prevent “branding” of the services as only for people with HIV and to prevent stigma and discrimination after the home visit. They also believed visiting all the homes could promote respect for the health care workers:

“*If they choose which areas/homes to visit they will lose respect and most people will not respect them at all so it’s better to visit homes without discrimination*”. (22-year-old male partner)

When participants were asked how to initiate discussion with male partners of clients at the home without causing problems, they highlighted the need to be professional, clear about the purpose of the visit, confidential, and tactful.

“*First, you have to know how to approach somebody, and the way you present yourself in your opinions that you can use while with them until they accept your message, and keep it a secret before you leave their door*”. (28-year-old male service provider)

After securing permission to conduct the visit the participants indicated that perhaps it would be beneficial if the health worker initiated the discussion with male partners by talking about broad issues before getting into specifics of HIV. They explained that the health workers are making the visit to provide support, so there are other issues that may be affecting individuals.

“*He should start by telling people all the general benefits and later start advising us. He shouldn’t be specific to the point. He should say the advantage of seeking treatment in case you have the disease. He should tell them to go for the test whether they are infected or not. He should also discuss the advantages of visiting clinics that deal with pregnancy*”. (22-year-old pregnant woman)

## Discussion

Our study highlights the perspectives of HIV-positive pregnant women, male partners, and community service providers on barriers and facilitators to HIV status disclosure, as well as approaches that could be used to assist pregnant couples in rural Nyanza Kenya to safely disclose their HIV status.

The findings suggest that fears of abuse, disharmony in the couple relationship, and stigma were significant barriers to HIV disclosure among pregnant couples. While fears of abuse [8,42] and stigma [43,44] are well documented barriers to HIV disclosure, the findings regarding couple relationship factors are less well known. These findings underscore the need for interventions that take into account couple relationship factors to facilitate safe HIV disclosure. These interventions could include approaches to promote relationship building skills, and communication strategies, especially about HIV testing and disclosure [45].

We found that no one method of facilitated HIV disclosure will be appropriate for every pregnant woman and the contexts in which women live must be taken into consideration when HIV disclosure is being recommended. Inasmuch as facilitated disclosure in the home or at a health care facility were seen as acceptable approaches for facilitated disclosure for pregnant women and their male partners in this rural Kenyan setting, a number of potential disadvantages were identified for both approaches. Further the method used to assist clients with disclosure is also crucial for optimizing the benefits and preventing unintended social consequences. The findings of this study support the literature showing that individuals are motivated by a multiplicity of factors in their decisions regarding HIV testing [46] and disclosure of their HIV status [8,10,19,47]. Thus multiple strategies are warranted to promote HIV testing and safe disclosure to a partner, and improve linkage to HIV care [48].

Most of the participants did not favor an approach in which the health worker would disclose a person’s HIV-infected status to his/her partner. The participants seem to feel strongly that this would be a denial of their right to disclose their HV status to their partner themselves. This highlights the complexity of HIV disclosure and the value individuals place on the ownership of their private information. Testing for HIV together as a couple was by far the most preferred method, primarily because it provided an opportunity to remove the burden of disclosure on any one individual, thus no one can be blamed for infecting the other. It was felt that even individuals with a prior positive HIV diagnosis could be protected by this method as they could pretend that they were never given a HIV-positive diagnosis and re-test with their partners. While this method may provide a safety net for the participants, it may create an ethical dilemma for health workers who would be asked to re-test clients knowing that they are already infected with HIV.

We found that home-based couples HIV counseling and testing, which includes support for mutual disclosure, was seen as acceptable and feasible by health care providers, pregnant women, and their male partners. This aligns with previous research in sub-Saharan Africa showing that home-based counseling HIV testing (HBCT) is effective, feasible to deliver, and acceptable to the target populations [24,30,48]. However, the highly stigmatizing nature of HIV requires that these home-based programs take special measures to protect confidentiality [31,34,49-51]. Indeed, other research has shown that home-based HIV testing can carry additional risks such stigma [31,52] fear of receiving positive results [53] (especially discordant results), unwanted disclosure to other family members, and breach of confidentiality by health workers [21]. Key recommendations suggested by our participants to avoid these risks of home-based testing for pregnant couples, include the careful selection and training of health care workers, promotion/branding of the program as general pregnancy health not HIV, and strategies to reduce HIV stigma. Making appointments before conducting the home visit, building trust, and involving the community appear to be important for the success of such a program. These findings highlight factors that could serve as barriers to the effective implementation of HBCT programs targeting HIV infected pregnant women and their partners. A proposed home-based couples HIV counseling and testing approach, including facilitated disclosure, for pregnant women and male partners—based on the findings of this study—is presented in Figure 1.

Providing HIV testing in homes could be used to solicit men’s participation in ANC and promote HIV testing and disclosure. This has the potential to improve health outcomes, since pregnant women are more likely to adhere to PMTCT care if their male partners are involved in ANC and HIV testing [12,54]. A recent study conducted by Roxby and colleagues involving 148 HIV-infected pregnant women who reside in rural Kenya also emphasized the importance of implementing strategies that will foster HIV testing among male partners of pregnant women [15]. Promoting couples testing for pregnant couples in PMTCT programs could improve health outcomes for women, children and partners.

Although this research contributes to the literature regarding measures that could promote HIV disclosure among couples, it has limitations. First, participants in this study reported higher levels of partner disclosure (75%) than documented in similar settings [15]. This is due in part to the way in which participants were recruited for the study. In particular, the men had to already know about the HIV-positive status of their female partners to be eligible for this study, to protect against unwanted disclosure and adverse consequences due to participation in the research. Second, the study did not include participants who do not use health care facilities, and are likely to be less informed and thus may have different perspectives on PMTCT and facilitated HIV disclosure.

However, the in-depth examination of the perspectives of HIV-positive pregnant women, male partners, and service providers is an important strength that we believe outweighs these limitations. Our study underscores the need to provide clients with different options for HIV disclosure, as no single method of disclosure will be suitable for everyone. Therefore, health workers must consider the social contexts in which pregnant women and their partners live when recommending HIV disclosure in order to reduce the effects of unintended consequences. These findings can inform the design and implementation of programs geared at promoting couples HIV counseling and testing, including support for mutual disclosure, among pregnant women and partners, especially in the home setting.

## Conclusions

HIV testing and counseling for couples was viewed as an acceptable strategy that would support mutual HIV status disclosure. Home-based couples HIV counseling and testing for pregnant women and their male partners in this and similar settings may provide an opportunity to increase uptake of antenatal HIV testing, increase men’s involvement in PMTCT, and improve HIV disclosure among partners, all of which collectively work to reduce vertical and horizontal HIV transmission. Future studies should assess the effectiveness of home-based couples-based HIV counseling and testing programs in improving health behaviors such as linkage to care, adherence among pregnant women and their partners, as well as on perinatal transmission rates.

## Competing interests

The authors declare that they have no competing interests.

## Authors’ contributions

MW conducted the qualitative coding and data analysis and drafted the manuscript. AH participated in data analysis and helped to draft the manuscript. ZK trained and supervised the qualitative research team in Kenya, contributed to data analysis, and helped to draft the manuscript. JT conceptualized the study, supervised data collection, participated in data analysis, and helped to draft the manuscript. All authors read and approved the final manuscript.

## Pre-publication history

The pre-publication history for this paper can be accessed here:

http://www.biomedcentral.com/1471-2458/13/1115/prepub
